# Stoichiometrical regulation of soil organic matter decomposition and its temperature sensitivity

**DOI:** 10.1002/ece3.1927

**Published:** 2016-01-09

**Authors:** Nianpeng He, Guirui Yu

**Affiliations:** ^1^ Key Laboratory of Ecosystem Network Observation and ModelingInstitute of Geographic Sciences and Natural Resources ResearchChinese Academy of SciencesBeijing100101China

**Keywords:** C:N ratio, heterotrophic respiration, kinetic models, mineralization, *Q*_10_, substrate quality, warming

## Abstract

The decomposition of soil organic matter (SOM) can be described by a set of kinetic principles, environmental constraints, and substrate supply. Here, we hypothesized that SOM decomposition rates (*R*) and its temperature sensitivity (*Q*_10_) would increase steadily with the N:C ratios of added substrates by alleviating N limitation on microbial growth. We tested this hypothesis by investigating SOM decomposition in both grassland and forest soils after addition of substrates with a range of N:C ratios. The results showed that Michaelis–Menten equations well fit the response of *R* to the N:C ratio variations of added substrates, and their coefficients of determination (*R* ^2^) ranged from 0.65 to 0.89 (*P *<* *0.01). Moreover, the maximal *R*,* Q*_10_, and cumulative C emission of SOM decomposition increased exponentially with the N:C ratios of added substrates, and were controlled interactively by incubation temperature and the N:C ratios of the added substrates. We demonstrated that SOM decomposition rate and temperature sensitivity were exponentially correlated to substrate stoichiometry (N:C ratio) in both grassland and forest soils. Therefore, these correlations should be incorporated into the models for the prediction of SOM decomposition rate under warmer climatic scenarios.

## Introduction

Soil is the largest carbon (C) sink in terrestrial ecosystems (IPCC, 2007), and decomposition of soil organic matter (SOM) is positively correlated with temperature (Kirschbaum 1995; Sierra et al. 2011). Understanding the relationship between climatic change and SOM decomposition is necessary in order to predict changes in soil C storage under warmer climatic scenario (Kirschbaum 1995). In recent decades, the temperature sensitivity of SOM decomposition (*Q*_10_, the factor by which the decomposition rate increases for every 10‐degree rise in the temperature) has been widely used to explore the responses of SOM decomposition to diurnal and/or seasonal temperature changes. However, a consensus has not yet reached among researchers on the mechanisms that control decomposition processes and spatial variations in *Q*_10_ (Davidson and Janssens 2006; Conant et al. 2011; Wagai et al. 2013).

Understanding the influences of substrate supply (or substrate availability) on *Q*_10_ is important for predicting the fate of SOM in a warmer world (Hartley et al. 2006; Conant et al. 2011). As reviewed by Davidson and Janssens (2006), the processes of SOM decomposition may be well described by a set of kinetic principles and environmental constraints. The influences of substrate availability on enzyme‐catalyzed reactions are often described using the Michaelis–Menten kinetics (*R *= *R*_max_ × *S*/(*K*_m_ + *S*) (Michaelis and Menten 1913; Davidson and Janssens 2006; Davidson et al. 2012). In this equation, *S* is the substrate availability, *R*_max_ is the maximum decomposition rate, and *K*_m_ is the Michaelis–Menten constant. *K*_m_ represents the substrate concentration at which decomposition rate is equal to half of *R*_max_. When decomposition substrates are abundant, the importance of *K*_m_ becomes trivial and *R*_max_ under specific temperature may determine the decomposition rate. Therefore, *R*_max_ is expected to increase with the external substrate addition, if *S* is abundant and the temperature is optimal for enzyme activity (Davidson and Janssens 2006; Gershenson et al. 2009).

The stoichiometry (e.g., nitrogen [N]:C ratio) of added substrates exerts an important influence on SOM decomposition by altering the relative availability of C‐ and N‐fixing soil microbes (Manzoni et al. 2010; Billings and Ballantyne 2013; Hartman and Richardson 2013; Weedon et al. 2013; Leifeld and von Lutzow 2014). Disproportionate stoichiometry in added substrates results in certain elements becoming limiting for microbial growth, thereby affecting SOM decomposition (Chapman 1997; Fierer et al. 2005). In natural ecosystems, changes in the N:C ratios of new SOM inputs from ground litter and root exudates may regulate the responses of microbes (Drake et al. 2013). In an incubation experiment, He et al. (2013) reported that high N:C ratios of added substrates significantly enhance *Q*_10_ due to the mitigation of N limitation. However, the assumption that *Q*_10_ increases predictably with the substrate N:C ratios has not been proven experimentally.

Here, we hypothesized that increasing N:C ratios of added substrates would result in steady increases in *R*_max_ and *Q*_10_ by alleviating N limitation on microbial growth. To test this hypothesis, we conducted two independent experiments. In the experiment I, we investigated short‐term responses of *R*_max_ and *Q*_10_ to added substrates with eight different N:C ratios and six incubation temperatures in grassland and forest soils, through intensively measuring SOM decomposition rates. In the experiment II, we explored the long‐term influence of substrate N:C ratios on the cumulative SOM decomposition in grassland and forest soils over a 56‐days incubation period.

## Material and Methods

### Study sites

Soil samples were collected from long‐term experimental plots in a typical grassland and a typical forest. The long‐term experimental plot (43°33′01″N, 116°40′20″E) of the grassland was located in Inner Mongolia and was managed by the Inner Mongolia Grassland Ecosystem Research Station (IMGERS), Chinese Academy of Sciences. The region has a semi‐arid continental climate with a mean annual precipitation of 345 mm and a mean annual temperature of 1.1°C from 1980 to 2010. The chestnut soils (i.e., Calcic Kastanozems) are equivalent to Calcic‐orthic Aridisol, according to the US soil classification system (He et al. 2008). Soil texture is comprised by 70.0, 24.3, and 5.7% for sand (50–2000 *μ*m), silt (2–50 *μ*m), and clay (<2 *μ*m), respectively (He et al. 2009). IMGERS established the experimental plots in 1999 by fencing against free‐grazing. The vegetation in the experimental plots was dominated by such as *Leymus chinensis*,* Stipa grandis*, and *Cleistogenes squarrosa*.

The long‐term experimental plots (39°57′82″ N, 115°25′68″ E) of the forest were located in Dongling Mountains and were managed by the Beijing Forest Ecosystem Research Station, Chinese Academy of Sciences. The region has a temperate continental monsoon climate with a mean annual temperature of 4.8 °C and annual precipitation of 612 mm from 1985 to 2010. The soils were classified as forest brown and cinnamon soils (Hou et al. 2006), where soil fractions are comprised by 77.5, 20.5, and 2.5% for sand (50–2000 *μ*m), silt (2–50 *μ*m), and clay (<2 *μ*m), respectively. The vegetation in the experimental plot is dominated by *Quercus liaotungensis* and *Betula dahurica,* which have been protected from intensive human disturbance since the 1950s (Li and Ma 2003).

### Soil sampling

Soil samples were collected at the end of July 2011. Four 2 × 2 m sampling quadrats were established in the grassland and forest plots (each about 100 × 100 m), respectively. Quadrats were placed randomly within the plots, and were separated from each other by at least 15 m. Soil samples (0–20 cm depth) were collected from 15 points within each quadrat using a 4‐cm‐diameter auger. Totally, four composite soil samples (>10 kg) were obtained from each plot and hand‐cleared off roots and visible organic debris, and then, approximately 100 g of each soil sample was air‐dried in a ventilation room for analyses of soil properties (e.g., C, N, and pH). The remaining soils were stored at 4 °C. Soil organic C (SOC, %) was measured using the modified Mebius method (Nelson and Sommers 1982). Total soil N (%) was measured using the modified Kjeldahl wet digestion procedure (Gallaher et al. 1976) and a 2300 Kjeltec Analyzer Unit (FOSS, Sweden). Soil pH was determined by a pH meter placed in soil mixed with distilled water at a ratio of 1:2.5. Selected soil properties are shown in the Table S1.

### Laboratory treatment and incubation

Soil water‐holding capacity (WHC, %) was measured using a simple substitute for soil core method (He et al. 2013). In brief, soil samples were placed in a dense screen mesh and soaked into distilled water for about 12 h. Samples were then placed in a dark, unvented cabinet for about 8 h. Samples were then weighed before and after drying for 48 h in an over at 105 °C for WHC determination.

The experiment had six incubation temperatures (5, 10, 15, 20, 25, and 30 °C) using eight different substrates with a range of N:C ratios (Table 1). To obtain an N:C ratio gradient, we collected aboveground litter, a mixture of grass leaves, and the leaves of *Medicago falcata* L. (a legume species) at the grassland plot. Similarly, we collected litter, a mixture of tree leaves, and the leaves of *Sophora japonica* L (a legume species) at the forest plots. Because of the difficulty to find natural litter with different C:N ratios but a similar soluble C content, we used the collected materials to create litter mixtures that fell along a C:N gradient. To accomplish this, air‐dried samples were ground in a high‐speed mill and sieved through a standard screen (40 mesh or 0.63 mm) to homogenize them. Thus, different ratios of litter, mixed leaves, and Leguminous leaves were mixed to obtain eight substrates with different N:C ratios (Table 1). The C content of the substrates was similar (44.0–45.1% in grassland and 43.1–44.4% in forest), but the substrates differed in N content, resulting in different N:C ratios (0.043–0.117 for the grassland and 0.054–0.075 for the forest). We did not consider the differences in lignin content and micro‐nutrients between different leaf and litter types, even though the presence of such differences may result in an inconsistent C quality across the different treatments (Schreeg et al. 2013).

**Table 1:** The N:C ratios of substrates added to grassland and forest soils

Grassland	Added substrates				Forest	Added substrates			
Abr.	Mixed ratio^a^ (L:M:G)	N content (g·kg^−1^)	C content (g·kg^−1^)	N:C	Abr.	Mixed ratio^b^ (L:M:G)	N content (g·kg^−1^)	C content (g·kg^−1^)	N:C
G0	0:0:0	0	0	0	F0	0:0:0	0	0	0
G1	1:0:0	19.46 ± 0.05	450.70 ± 0.21	0.043	F1	1:0:0	23.42 ± 0.14	431.08 ± 0.49	0.054
G2	4:1:0	25.88 ± 0.02	448.58 ± 0.25	0.058	F2	4:1:0	25.42 ± 0.11	433.94 ± 0.51	0.059
G3	3:2:0	32.31 ± 0.03	446.46 ± 0.29	0.072	F3	3:2:0	27.42 ± 0.08	436.81 ± 0.54	0.063
G4	0:1:1	35.52 ± 0.04	445.40 ± 0.31	0.080	F4	0:1:1	28.42 ± 0.07	438.24 ± 0.55	0.065
G5	0:2:3	38.73 ± 0.06	444.35 ± 0.33	0.087	F5	0:2:3	29.42 ± 0.07	439.67 ± 0.57	0.067
G6	0:1:4	45.16 ± 0.08	442.23 ± 0.37	0.102	F6	0:1:4	31.42 ± 0.07	442.53 ± 0.59	0.071
G7	0:0:1	51.58 ± 0.11	440.11 ± 0.42	0.117	F7	0:0:1	33.43 ± 0.09	445.40 ± 0.62	0.075

a L, litter; G, mixed grass leaves; and M, leaf of *Medicago falcata* L. (a legume species) in the grassland.

b L, litter, G, mixed tree leaves, and M, leaf of *Sophora japonica* L. (a legume species) in the forest.

#### Experiment I

The experiment I was designed to test how the N:C ratios of added substrates influence the short‐term responses of SOM decomposition under abundant substrate availability. Fresh soil samples (40 g) were placed into incubation bottles and adjusted to 60% WHC. Soil samples were placed in an incubator set at 20 °C and 80% humidity for 4 days, and then were incubated at different temperatures (5, 10, 15, 20, 25, and 30 °C) for 3 days. We measured soil respiration rates as the basal data prior to substrate addition. We then added 0.4 g the external substrates for each substrate. The substrates were mixed evenly with soil samples by shaking. Five replicate samples were prepared for each treatment, for a total of 480 samples (five replicates × eight substrates × six incubation temperatures × two soil types). During the 1‐day incubation experiment, the rates of SOM decomposition (*R*) were measured 11 times, at 0, 0.167, 0.33, 0.5, 1, 2, 4, 8, 12, 18, and 24 h, where *R* were transferred from the changes in CO_2_ concentration in the incubation bottle and other parameters (see details in the following section). We conducted the experiment using fed‐batch incubation (30 times), due to limitations imposed by the time required to measure *R*.

#### Experiment II

This experiment II was designed to test whether higher N:C ratios promote SOM decomposition over long periods. Fresh soils (40 g) were placed into incubation bottles and adjusted to 60% WHC. The samples were placed in an incubator set at 20 °C and 80% humidity for 4 days. Then, *R* were measured as basal data. The eight substrates prepared for each soil type were added to the soil samples and mixed evenly by shaking, as experiment I did. During the 56‐day incubation experiment, *R* were measured 14 times on days 0, 1, 2, 3, 4, 5, 6, 7, 14, 21, 28, 35, 42, 49, and 56.

To enhance the speed and accuracy of *R* measurement, we developed a new auto‐sampling and analyzing system (He et al. 2013), which was modified from the continuous gas flow system of Cheng and Virginia (1993). In practice, *R* in each bottle was calculated from the slope of CO_2_ concentration and specific transforming factors (see the details in He et al. 2013), as follows:

(1) $$R=\frac{C\times V\times \alpha \times \beta }{m}$$

where *R* is the SOM decomposition rate (*μ*gC·g^−1^·hour^−1^), *C* is the slope of CO_2_ concentration; *V* is the volume of the incubation bottle and gas tube, *m* is the soil weight, *α* is the transformation coefficient of CO_2_ mass (12/22.4), and *β* is the transformation coefficient of time (He et al. 2013).

### Calculations and statistical analysis

The Michaelis–Menten kinetics equation (Eq. (2)) was used to investigate the effect of N:C stoichiometry of the added substrates on *R* (Michaelis and Menten 1913; Gershenson et al. 2009).

(2) $$R=\frac{R_{\max}\times \left[S\right]}{K_{m}+\left[S\right]}$$

where *R*_max_ represents the maximum decomposition rate achieved by the system at different substrate N:C ratios. *S* is the N:C ratios of added substrates. *K*_m_ is the Michaelis constant representing the substrate concentration at which *R* arrives at half of *R*_max_.

*Q*_10_ in experiment I was calculated using the following exponential equations:

(3) $$R=A\times {\text{exp}}^{B\times T}$$

(4) $$Q_{10}={\text{exp}}^{10\times B}$$

where *R* is the SOM decomposition rate (*μ*gC·g^−1^·hour^−1^), *T* is the temperature (°C), *B* is the response efficiency or *R* to changing temperature, and *A* is the C quality index.

In this study, we defined the C quality of substrates as the relative rate to *R*. The C quality of SOM decomposition was equal to *A* (*μ*gC·g^−1^·day^−1^) in Eq. (3), which corresponds to the y‐intercept of the first‐order exponential equation relating *R* to temperature. Several previous studies have described substrate C quality in a similar manner (e.g., Bosatta and Agren 1999; Fierer et al. 2005). Parameter *A* provides an index of the overall C quality of substrates (availability and lability) that are catabolized by decomposer organisms at a given time.

One‐way analysis of variance (ANOVA) was used to explore whether the *Q*_10_ values differed significantly among substrates. Linear and curve regressions were used to identify the trend of *Q*_10_ changes and cumulative SOM decomposition with changes in the N:C ratios of the substrates. The best‐fit equations were identified using the Akaike information criterion (AIC) and the Bayesian information criterion (BIC) (Aho et al. 2014). Data are presented as means ± standard deviation. Differences were considered to be significant when *P *<* *0.05. All analyses were conducted using SPSS statistical software (v. 13.0, SPSS, Chicago, IL) and Sigmaplot (v. 10.0, Sigmaplot, Oregon, Corvallis).

## Results

### Effects of the N:C ratios of added substrates and temperature on short‐term SOM decomposition

In the experiment I, *R* increased immediately after substrates were added, and the maximum decomposition rate was observed (*R*_max‐obs_) 0.5–2 h later, and then *R* decreased gradually. The *R*_max‐obs_ ranged from 1.3 to 10.5 *μ*gC·g^−1^·hour^−1^ in grassland soil and from 2.4 to 6.4 *μ*gC·g^−1^·hour^−1^ in forest soil. Both the N:C ratios of the added substrates and the incubation temperature had significant effects on *R*_max‐obs_, with apparent interactive effects (Table 2).

**Table 2:** Univariate analysis of the observed maximum decomposition rate (*R*_max‐obs_) with respect to N:C ratio of substrate and temperature

	Grassland soil		Forest soil	
	*F*	*P*	*F*	*P*
N:C ratio of substrate (S)	7997.7	<0.0001	4164.9	<0.0001
Temperature (T)	4588.4	<0.0001	1804.4	<0.0001
S × T	330.8	<0.0001	126.5	<0.0001

**Figure 1:**
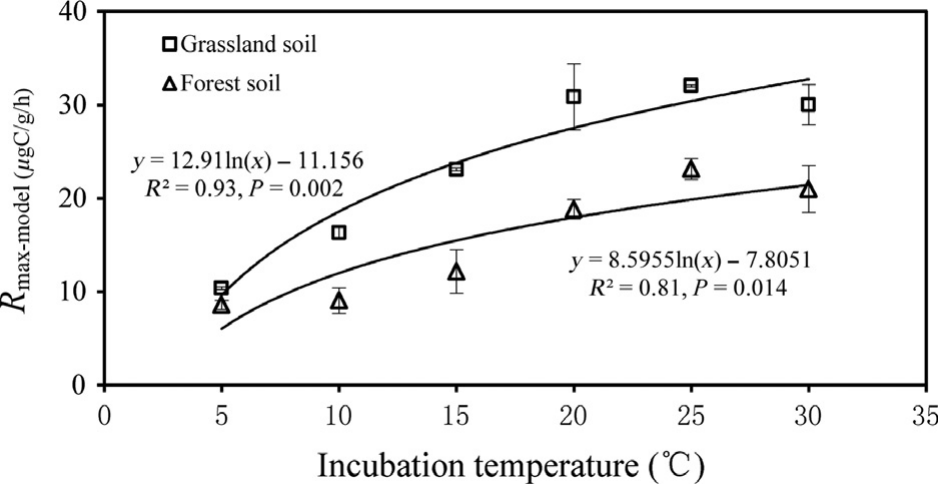
Changes in the maximum decomposition rate derived from the of Michaelis–Menten kinetic equation (*R*_max‐model_) with incubation temperature. A logistic equation well depicted the relationship between the *R*_mas‐model_ and temperature. The *R*_mas‐model_ was dependent on the N:C ratio of substrates.

The responses of microbial respiration to the different N:C ratios of the added substrates were described by the Michaelis–Menten kinetics equations well, and the coefficient of determination (*R* ^2^) ranged from 0.65 to 0.89 (*P *<* *0.001 for all) (Fig. S1 and Table S2). Moreover, the maximum decomposition rate derived from the Michaelis–Menten equations (*R*_max‐model_) increased logarithmically with incubation temperature in both grassland (*R* ^2^ = 0.93, *P *=* *0.002) and forest soils (*R* ^2^ = 0.81, *P *=* *0.014) (Fig. 1).

**Figure 2:**
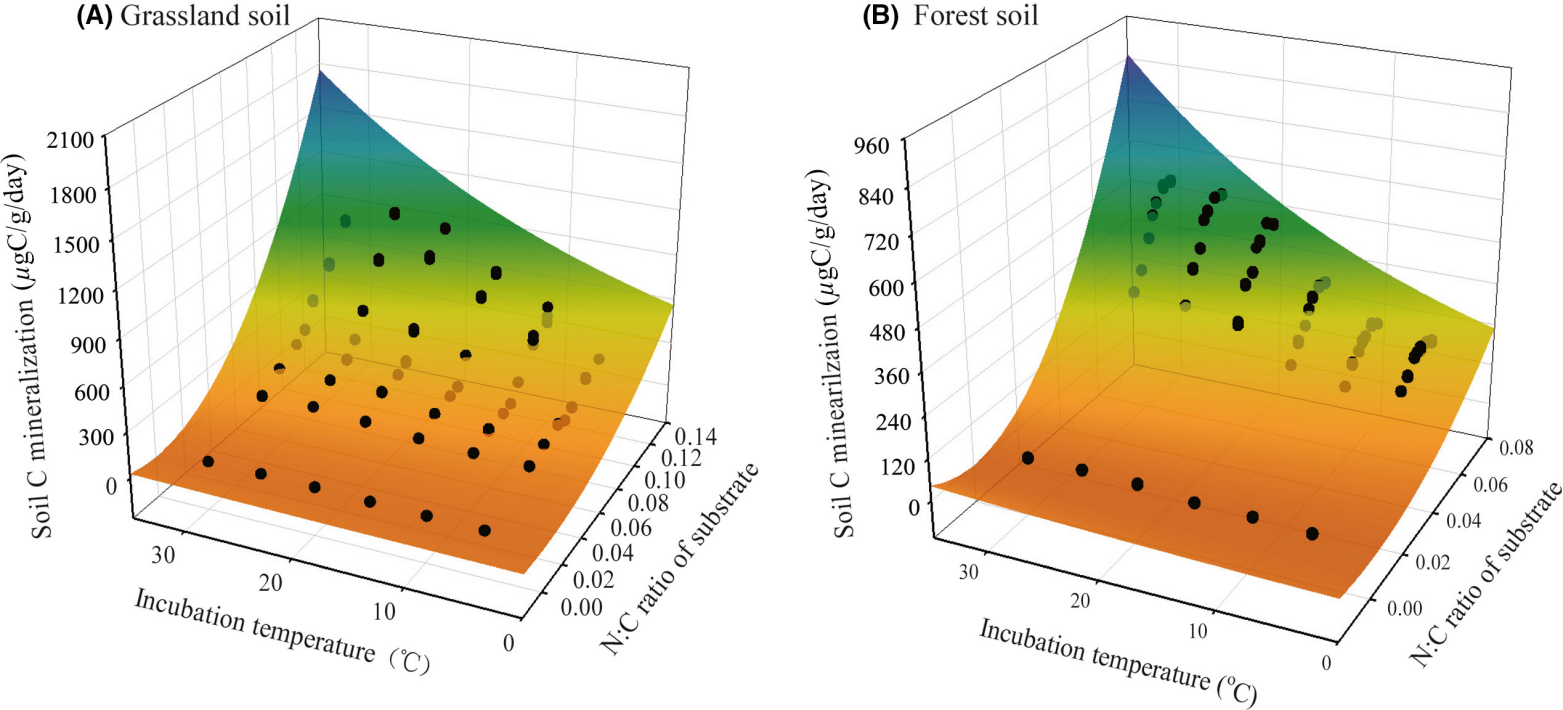
Dependence of soil organic matter decomposition rates on the N:C ratio of the substrates and incubation temperature in grassland soil (A) and forest soil (B). Data were derived from 14 time measurements during a 1‐days incubation experiment.

The N:C ratio of the added substrates and incubation temperature influenced cumulative SOM decomposition in the 1‐day incubation experiment, being well fit with the model (z = a × *x* ^2^ × e^by^) (*R* ^2^ = 0.89, *P *<* *0.001 for grassland soil; *R* ^2^ = 0.89, *P *<* *0.001 for forest soil) (Fig. 2).

**Figure 3:**
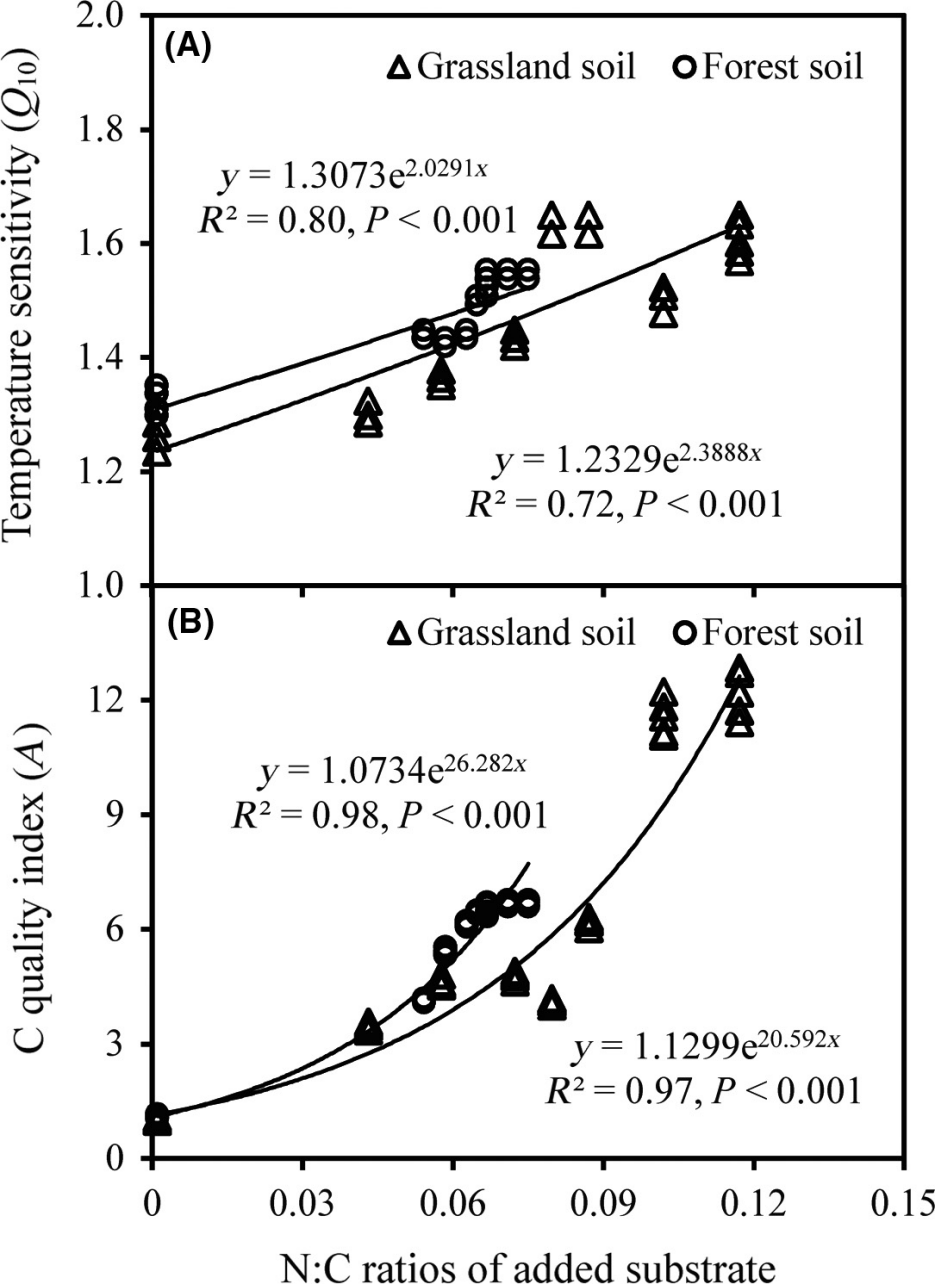
Influences of the N:C ratio of the added substrates on the temperate sensitivity (*Q*_10_) of soil organic matter decomposition and C quality indices. Data were derived from 14 time measurements during a 1‐days incubation experiment.

The *Q*_10_ differed significantly between the soils with different substrate N:C ratios, both for grassland (F = 276.9, *P *<* *0.001) and forest (F = 195.9, *P *<* *0.001). As expected, *Q*_10_ was exponentially related to the N:C ratio of added substrates (*R* ^2^ = 0.72, *P *<* *0.001 for grassland soil; *R* ^2^ = 0.80, *P *<* *0.001 for forest soil) (Fig. 3). The C quality index (A) also exponentially increased with increasing N:C ratios of the added substrates (Fig. 3).

### Effect of the N:C ratios of added substrates on long‐term SOM decomposition

**Figure 4:**
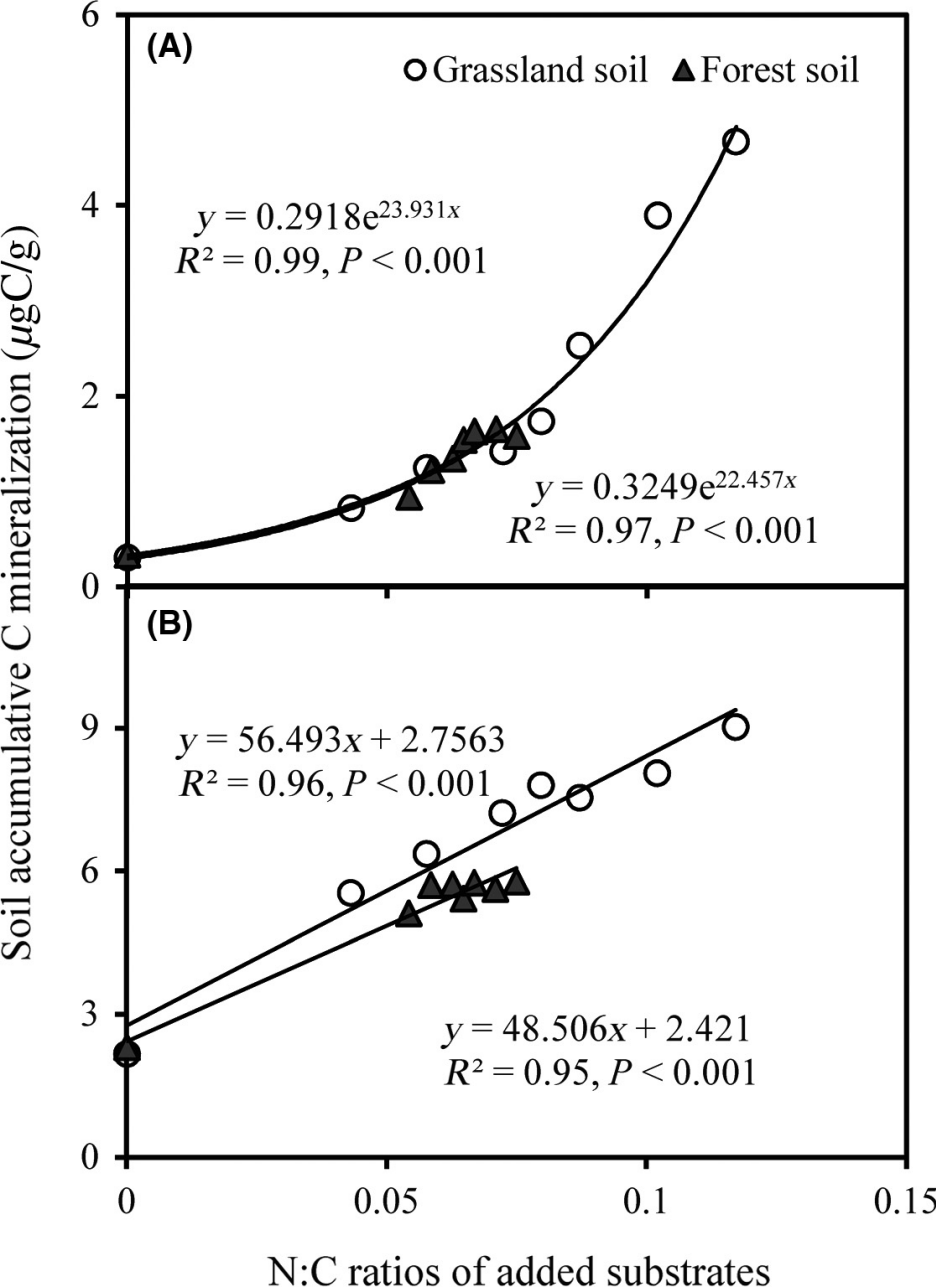
Changes in the accumulation of soil organic matter decomposition with the N:C ratio of added substrates during the first week (B) and the entire 7‐week experimental period (B). Soil samples were incubated at 20°C and at 60% soil water‐holding capacity

SOM decomposition over 56‐day incubation was influenced significantly by the N:C ratios of the added substrates (Fig. S2). During the initial phase (7 days), cumulative soil C emission increased exponentially with the N:C ratios of added substrates (*R* ^2^ = 0.99, *P *<* *0.001 for grassland soil; *R* ^2^ = 0.97, *P *<* *0.001 for forest soil) (Fig. 4A). Over the total incubation period (56 days), cumulative SOM decomposition was linearly related to the N:C ratios of the added substrates in grassland (*R* ^2^ = 0.96, *P *<* *0.001) and forest soils (*R* ^2^ = 0.95, *P *<* *0.001) (Fig. 4B).

## Discussion

In the present study, the N:C ratios of added substrates had a strong influence on SOM decomposition in grassland and forest soils. The N:C ratios of added substrates affected the response intensity and duration of SOM decomposition. In general, SOM decomposition tended to respond 10–20 min after substrate addition irrespective of N:C ratios. The rapid response of SOM decomposition to the addition of substrates is consistent with those reported by Jones and Murphy (2007), who used sugar as the added substrates. The rapid response of SOM decomposition rates to additional substrate may be partially attributed to the large degree of functional redundancy within soil microbial communities (Leifeld and von Lutzow 2014). Furthermore, SOM decomposition rates (or *R*_max_) increased with N:C ratio of added substrates, because the substrates with relatively higher N content may meet the N demand of microbes (Leifeld and von Lutzow 2014). Given the rapid responses of SOM decomposition rates to added substrates, grinding‐homogenization and evenly mixing of decomposition substrates are very important for similar incubation experiments. Incomplete mixing or uneven distribution of substrate therefore may be to some extent contributed to the observed variation between samples in this study.

The *Q*_10_ exhibited a significant exponential increase with increasing substrate N:C ratios in both grassland and forest soils. This supported our hypothesis that *Q*_10_ increased steadily with increasing substrate N:C ratios due to alleviation of N limitation on microbial growth. These findings are consistent with our previous study (He et al. 2013), in which *Q*_10_ increased significantly with substrate addition and *Q*_10_ was highest after the addition of leguminous substrate. Furthermore, Gershenson et al. (2009) reported that the addition of external substrates (C resources) may significantly enhance *Q*_10_. The observed exponential relationship between *Q*_10_ and the N:C ratios of added substrates likely result from substrate saturation eliminating the canceling effect of *K*_m_ on temperature sensitivity over a short time. In the Michaelis–Menten equation, both the *R*_max_ and *K*_m_ are positively temperature dependent. At high substrate concentration (S), *K*_*m*_ does not significantly contribute to *R,* and *R* is determined by the response of *R*_max_ to temperature. Furthermore, when *S* is abundant, the response of *R*_max_ to temperature determines *R* (Billings and Ballantyne 2013). Therefore, both *R*_max_ and *Q*_10_ increase with temperature under abundant substrates until an optimum temperature is reached (Atkin and Tjoelker 2003; Gershenson et al. 2009).

The C quality index of substrates (A) increased exponentially with the N:C ratio, which indicates that substrates with a higher N:C ratio are decomposed relatively easily by soil microbes (Fierer et al. 2005; Melillo et al., 1984). Our findings are not necessarily contrary to the traditional idea that recalcitrant substrates (or low N substrates) have higher Q_10_ than other substrates (Fierer et al. 2005; Davidson and Janssens 2006; Craine et al. 2010)*,* or to the carbon‐quality temperature hypothesis, which states that *Q*_10_ is negatively related to the C quality of substrates (Bosatta and Agren 1999). These previous findings are correct for SOM decomposition without newly SOM input or under the condition of labile SOM depleted increasingly. As discussed above, under the scenarios of external SOM input (or abundant labile SOM), newly input SOM (irrespective of N:C ratios) has a significantly positive influence on *Q*_10_, and the Michaelis–Menten equation may well depict the response of *R* to added substrates by eliminating the canceling effect of *K*_m_ on the apparent *Q*_10_ over a short period of time (Fig. S1 and Table S2).

*Q*_10_ in situ depends to some extent on substrate availability and quality (Davidson and Janssens 2006; von Lutzow and Kogel‐Knabner 2009). This study shows that *Q*_10_ and cumulative SOM decomposition increased with the N:C ratio of added substrate. Dias et al. (2013) found that N‐driven changes in plant communities influence leaf‐litter traits, and may alter the process of SOM decomposition. Therefore, changes in plant communities and the N:C ratios of new SOM inputs are expected to influence SOM stability and storage (Drake et al. 2013; He et al. 2013). In other words, changes in the quantity, quality, and availability of substrates at different temporal and spatial scales in natural ecosystems may contribute to the large variability in the observed *Q*_10_ of soil respiration in natural ecosystems.

## Conclusions

The stoichiometry (N:C) of added substrates has an important influence on the responses of SOM decomposition to temperature, which is consistent with the principles of kinetic theory and substrate constraints. The N:C ratios of added substrates and incubation temperature interactively influenced the response time and response intensity of SOM decomposition, as reflected by an exponential increase in both *Q*_10_ and *R*_max_ with substrate N:C ratio. Substrate stoichiometry has a steady effect on SOM decomposition and *Q*_10_ in both grassland and forest soils when substrate is not limiting. This study highlights the potential impact of substrate stoichiometry on SOM decomposition under warming scenarios.

## Conflict of Interest

None declared.

## Supporting information


**Table S1.** Selected properties of the two soils used in the incubation experiments.
**Table S2.** The statistics of the Michaelis‐Menten kinetics equations between soil organic matter decomposition rates and the N:C ratios of added substrates in grassland soil and forest soil.
**Figure S1.** The fitted functions of Michaelis‐Menten equations between soil organic matter decomposition rates (*R*) and the N:C ratios of added substrates in grassland soil (A) and forest soil (B).
**Figure S2.** Accumulation of soil organic matter decomposition with time and added substrates.
