# Supplementary material for: Stoichiometrical regulation of soil organic matter decomposition and its temperature sensitivity
Source: Ecol Evol. 2016 Jan 9;6(2):620–7. doi: 10.1002/ece3.1927 (PMC4729244; doi:10.1002/ece3.1927)
Supplement: Supplementary file 1 — Table S1. Selected properties of the two soils used in the incubation experiments. Table S2. The statistics of the Michaelis‐Menten kinetics equations between soil organic matter decomposition rates and the N:C ratios of added substrates in grassland soil and forest soil. Figure S1. The fitted functions of Michaelis‐Menten equations between soil organic matter decomposition rates (R) and the N:C ratios of added substrates in grassland soil (A) and forest soil (B). Figure S2. Accumulation of soil organic matter decomposition with time and added substrates. [file ECE3-6-620-s001.docx]

**Title:** Substrate stoichiometry regulates soil organic matter decomposition and temperature sensitivity

Authors: He Nianpeng^*^, Yu Guirui

Key Laboratory of Ecosystem Network Observation and Modeling, Institute of Geographic Sciences and Natural Resources Research, Chinese Academy of Sciences, Beijing 100101, China

^*^Correspondent authors

Tel. 86-10-64889263

Fax. 86-10-64889432

E-mail: [henp@igsnrr.ac.cn](mailto:henp@igsnrr.ac.cn) (N.H.)

**Table S1** Selected properties of the two soils used in the incubation experiments

|  | Organic carbon  (g kg^–1^） | Total nitrogen  (g kg^–1^) | Total phosphorus  (g kg^–1^) | pH | water holding capacity  (WHC, %) |
| --- | --- | --- | --- | --- | --- |
| Grassland soil | 18.19 ± 0.49 | 1.72 ± 0.10 | 0.30 ± 0.01 | 7.66 ± 0.19 | 31.48 ± 0.87 |
| Forest soil | 15.68 ±1.27 | 3.11± 0.20 | 0.44 ± 0.02 | 7.07± 0.17 | 28.98 ± 1.13 |

**Table S2** The statistics of the Michaelis-Menten kinetics equations between soil organic matter decomposition rates and the N:C ratios of added substrates in grassland soil and forest soil.

|  | Grassland soil |  |  |  |  | Forest soil |  |  |  |
| --- | --- | --- | --- | --- | --- | --- | --- | --- | --- |
|  | *R*_max_ | R^2^ | F | *P* |  | *R*_max_ | R^2^ | F | *P* |
| 5°C | 10.38 | 0.857 | 42.9 | 0.0006 |  | 8.59 | 0.882 | 53.8 | 0.0004 |
| 10°C | 16.03 | 0.705 | 17.9 | 0.0058 |  | 9.12 | 0.859 | 44.0 | 0.0006 |
| 15°C | 23.05 | 0.647 | 13.8 | 0.0098 |  | 12.16 | 0.842 | 38.2 | 0.0008 |
| 20°C | 31.74 | 0.852 | 103.9 | 0.0012 |  | 19.13 | 0.889 | 58.4 | 0.0003 |
| 25°C | 32.05 | 0.748 | 21.8 | 0.0034 |  | 21.04 | 0.873 | 49.1 | 0.0004 |
| 30°C | 33.26 | 0.722 | 30.5 | 0.0080 |  | 21.48 | 0.880 | 57.0 | 0.0005 |

Michaelis-Menten kinetics equations represented as *R* = (*R*_max_× S ) / (*K*_m_ + S),

where *R*_max_ represents the maximum decomposition rate achieved at different substrate N:C ratios; S is the N:C ratios of added substrates, and *K*_m_ is the Michaelis constant, indicating the substrate concentration at which *R* is half of *R*_max_.

­­­­­

**
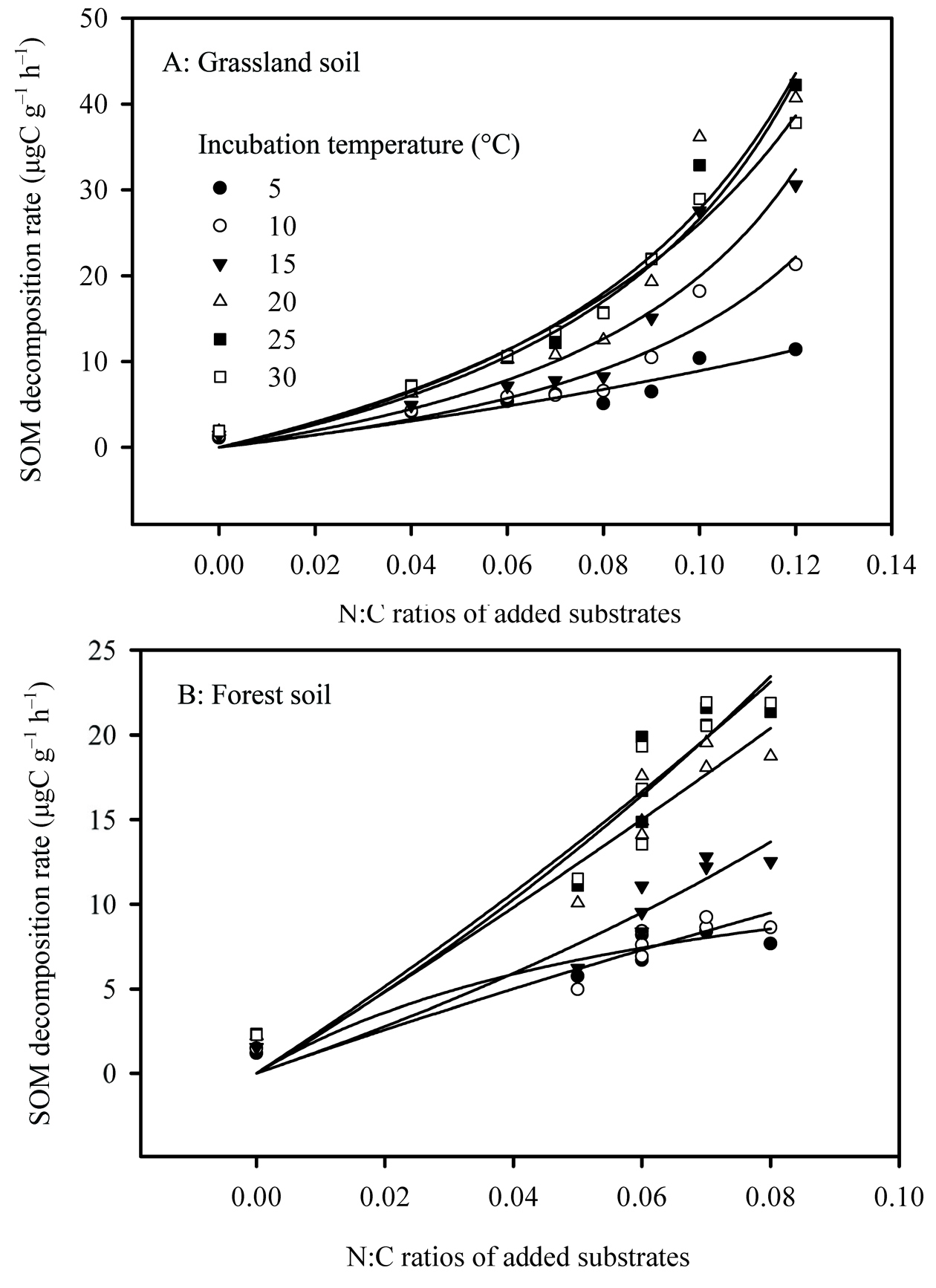
**

**Fig. S1** The fitted functions of Michaelis-Menten equations between soil organic matter decomposition rates (*R*) and the N:C ratios of added substrates in grassland soil (A) and forest soil (B). The values of *R* were the average of 14 measurements during a 1-d incubation experiment (See Table 1 for experimental treatments).


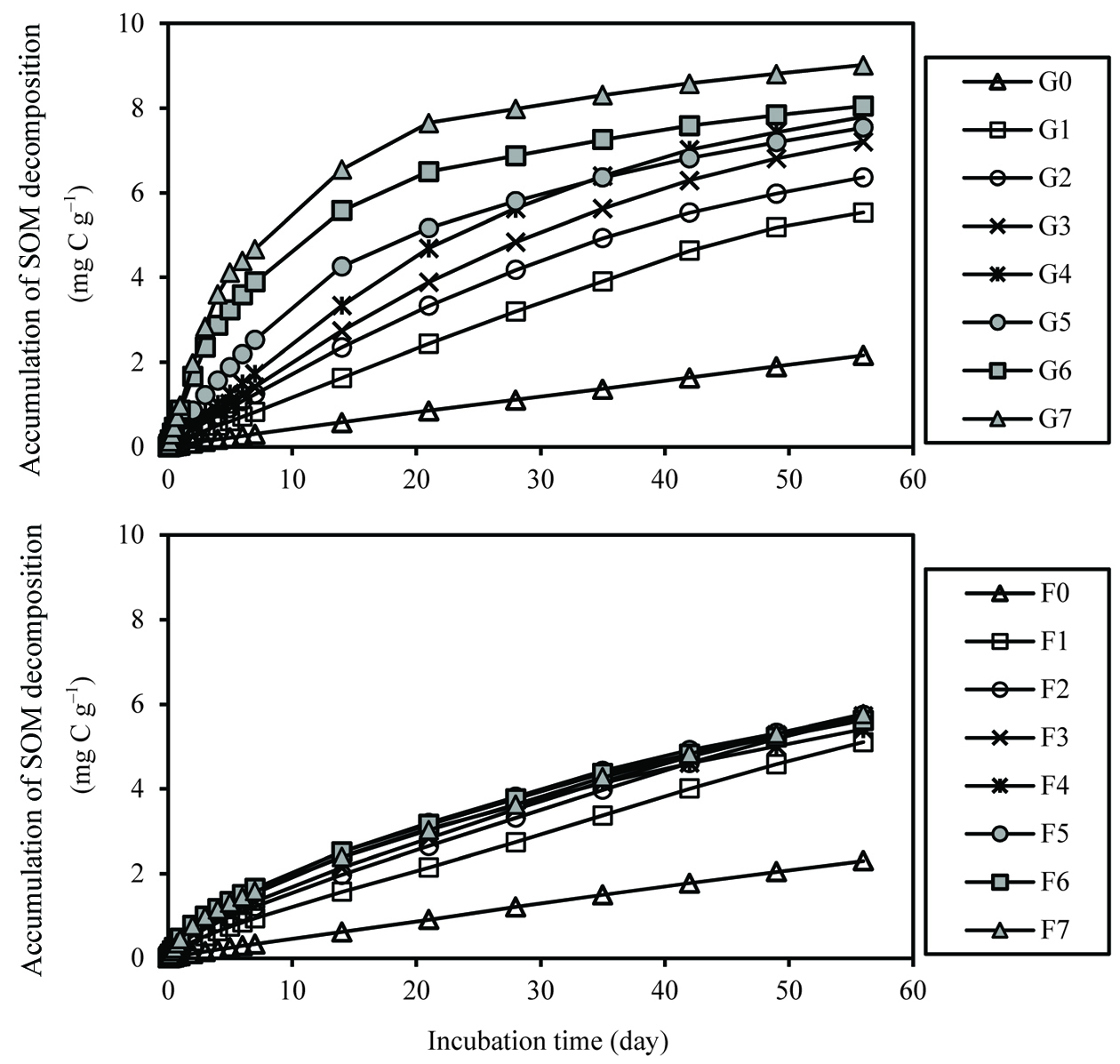


**Fig. S2** Accumulation of soil organic matter decomposition with time and added substrates. Soil samples were incubated at 20 °C and 60 % soil water holding capacity. (See Table 1 for experimental treatments)
